# Anatomical variations of cystic artery, cystic duct, and gall bladder and their associated intraoperative and postoperative complications: an observational study

**DOI:** 10.1097/MS9.0000000000001079

**Published:** 2023-07-10

**Authors:** Rohit Gupta, Anil Kumar, Chinniahnapalaya P. Hariprasad, Manoj Kumar

**Affiliations:** a Department of General surgery; b Department of Trauma Surgery and Critical Care, All India Institute of Medical Sciences, Patna, India

**Keywords:** anatomical variations, caterpillar hump, critical view of safety, laparoscopic cholecystectomy, postcholecystectomy syndrome, vasculobiliary injuries

## Abstract

Anatomical variations in the calots triangle encountered during laparoscopic cholecystectomy are not uncommon. Misidentification and misperception of these structures are the major cause of vasculobiliary injuries. This study was conducted to estimate the prevalence of anatomical variations of the cystic artery, cystic duct (CD), and gall bladder. This is the first study in India to access the rate of intraoperative and postoperative complications in anatomical variants compared to normal individuals.

**Patients and methods:**

It was a prospective observational study on patients undergoing laparoscopic cholecystectomy in the department of General Surgery at the tertiary center of India. The calculated sample size was 298. Variations of the cystic artery, CD, and gall bladder along with intraoperative and postoperative complications were noted. The comparative analysis of intraoperative and postoperative complications and a subgroup analysis between anatomical variants and normal patients were performed.

**Results:**

The most common variations were found in cystic arteries (16.8%). CD anomalies were present in 11.4% of patients, and gall bladder anomalies were the least common of all (5.4%). Intraoperative and postoperative complications were compared between patients with anatomical variations and normal anatomy. Intraoperative complications in patients with anatomical variations were significantly higher. Bile leak (15.7% vs. 6.4%) (*P*=0.01), haemorrhage (16.8% vs. 1.9%) (*P*-value <0.001), conversion to open (3 vs. 0 patients) (*P*-value =0.03). Subgroup analysis revealed a strong association between intraoperative haemorrhage and bile leak with cystic artery and CD anomalies, respectively.

**Conclusion:**

Cystic artery anomalies are the most common variations. Patients with anatomical variations had significant intraoperative and postoperative complications compared to patients with normal anatomy.

## Introduction

Highlights

- Vasculobiliary injuries are the major cause of morbidity in patients undergoing cholecystectomy, misinterpretation, and anatomical variations are the most common factors.
- The laparoscopic anatomy of the calots triangle is different and its assessment by preoperative and intraoperative investigations is difficult.
- This study provides insight about the operative anatomy of the calots triangle and possible anatomical variations of the cystic artery, cystic duct, and gall bladder.
- This is the first international study to compare intraoperative and postoperative complications among anatomical variants and normal individuals.
- Subgroup analysis revealed a strong association between intraoperative hemorrhage and bile leak with cystic artery and cystic duct anomalies, respectively.

Gall stones disease has become a common surgical problem, with loads of patients visiting the hospital for surgery on a daily basis. In recent era, laparoscopic cholecystectomy has been accepted as the gold standard technique, limiting the acceptance of open cholecystectomy^1^. Laparoscopic cholecystectomy has evolved significantly since its advent, like minimizing laparoscopic ports and use of three port techniques^2^. Variations in the anatomy are frequently encountered during the procedure, diagnosis of these variations preoperatively by routine investigations is a difficult task^3^. These vascular and biliary variations in the calots triangle contribute to the majority of intraoperative complications during laparoscopic cholecystectomy^4^.

Arterial anomalies are more frequent than biliary anomalies and can only be recognized after careful and meticulous dissection. The reported mortality rate because of injuries to blood vessels during laparoscopic cholecystectomy is about 0.02%^5^. Although the overall incidence of bile duct injury has declined in the past few decades, laparoscopic procedures still have a higher incidence of bile duct injury as compared to open cholecystectomy. Iatrogenic injuries of the bile duct account for serious complications during the procedure in around 0.3–0.5% of cases^6^. Until now, cadaveric dissections, laparoscopic ultrasonography, and various types of intraoperative cholangiography have been used to learn about vasculobiliary anatomy, but the figures quoted in the literature are variable. The conversion rate to open surgery because of vasculobiliary injury is ~0–1.9%^7^.

Knowledge of the possible variations will help prevent intraoperative and postoperative complications and decrease mortality and morbidity. No previous study has compared the intraoperative and postoperative complications between normal individuals and patients with anatomical variations. Therefore, this study was conducted with an objective to analyze the variations of the cystic artery, cystic duct (CD), and gall bladder and access the intraoperative and postoperative complications in patients with anatomical variations compared to normal individuals.

## Patients and methods

This study was a prospective observational study done in the department of General surgery at a tertiary center of India. All patients above 15 years of age undergoing laparoscopic cholecystectomy were included in the study. A total of 320 patients were assessed, patients with a history of upper abdominal surgery, frozen calots, laparoscopy converted to open due to a nonvascular cause, cases in which a critical view of safety could not be achieved, and patients unfit for general anesthesia were excluded. This study is in line with STROCSS guidelines^8^. Considering the previous study^9^, it was assumed that 27% of patients undergoing laparoscopic cholecystectomy have anatomical variations, the calculated sample size was 298 with a 5% level of significance and 95% confidence, considering the relative margin of error at 20%.

After preoperative evaluation and ultrasound of the upper abdomen, 310 patients underwent laparoscopic cholecystectomy. Preoperative antibiotic prophylaxis was given to all patients in the form of a single shot of cefaperazone-sulbactam (3rd generation cephalosporin) at the time of induction of anesthesia. Frozen calots was encountered in two patients, a critical view of safety could not be achieved in five patients, rest all 303 patients were assessed for extrahepatic biliary duct and vascular anatomy and their relations with each other. CD length was measured using Maryland tip span.

Normal anatomy was accepted as a single cystic artery lying posteriorly to the CD inside the calots triangle, a single CD of length between 2 and 4 cm, entering the common bile duct at an angle. A single gall bladder located at the cystic plate inside the gall bladder fossa, right side of the ligamentum teres was considered normal.

Intraoperative complications like bile leak due to injury and bleeding during the procedure were noted and compared between normal individual and anatomical variants. Intraoperative hemorrhage was defined as any unusual bleeding from an injured vessel during dissection of the calots triangle or separating the gall bladder from its fossa. Intraoperative bile leak was defined as any event of bile leak either from an injured duct, gall bladder rupture, or leak from the cystic plate due to an accessory duct. Intrahepatic gall bladder was defined as partial or complete internalization of the gall bladder inside the liver parenchyma. The need for drain insertion as per the discretion of the surgeon was noted. Intraoperative videos and photo recordings were made for cases with significant anomalies and reviewed by two different senior laparoscopic surgeons.

Postoperatively, all patients were evaluated for pain at 6 h, 24 h, 48 h of surgery and the time of discharge by using the visual analog scale with the same amount and type of analgesia. A nonsteroidal anti-inflammatory drug (Diclofenac) 75 mg was given twice daily as an analgesic. Length of hospital stay in days, any sign of port site infection at discharge and first follow-up visit were noted. During follow-up five patients were lost. A subgroup analysis for intraoperative and postoperative complications was performed among the remaining 298 patients with different types of anomalies.

**Figure:**
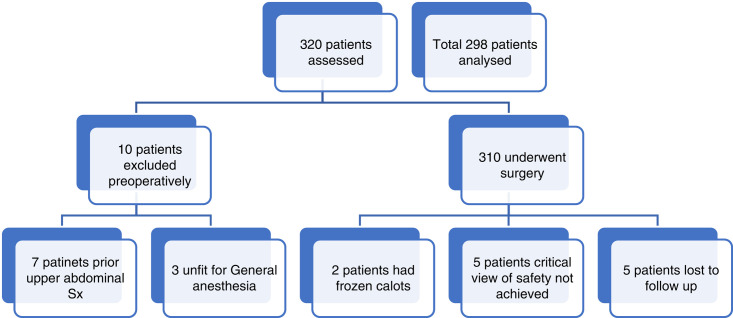


### Statistical analysis

All the data was collected in preformed data capture sheets and evaluated. Data was described in terms of range; mean±SD, median, frequencies (number of cases), and relative frequencies (percentages) as appropriate. For comparing categorical data, χ^2^ test was performed. A *P*-value of <0.05 was considered statistically significant. For nonparametric data, the Mann–Whitney *U* test was applied. For continuous data, an independent *t*-test was applied. A one-way ANOVA test was applied for subgroup analysis. All statistical calculations were done using SPSS (Statistical Package for the Social Science), a version 22 statistical program.

## Results

### Demographic and clinical profile

Out of 298 patients included in the study, the majority were from 2nd to 6th decade of life. The average age of the patients, who participated in this study was 41.3±13.3 years. The majority of patients were female (59.7%). BMI was segregated into underweight (1.3%), normal (35.2%), overweight (25.8%), and obese (37.2%). The most common presenting symptom was pain in the right upper quadrant (56.4%). No significant difference was found in the demographic and clinical profile between the two groups (Table 5).

### Prevalence of anatomical variations

During laparoscopic cholecystectomy of 298 patients, 68.1% had normal anatomy and anatomical variation was encountered in 31.9% of patients.

**Figure 1:**
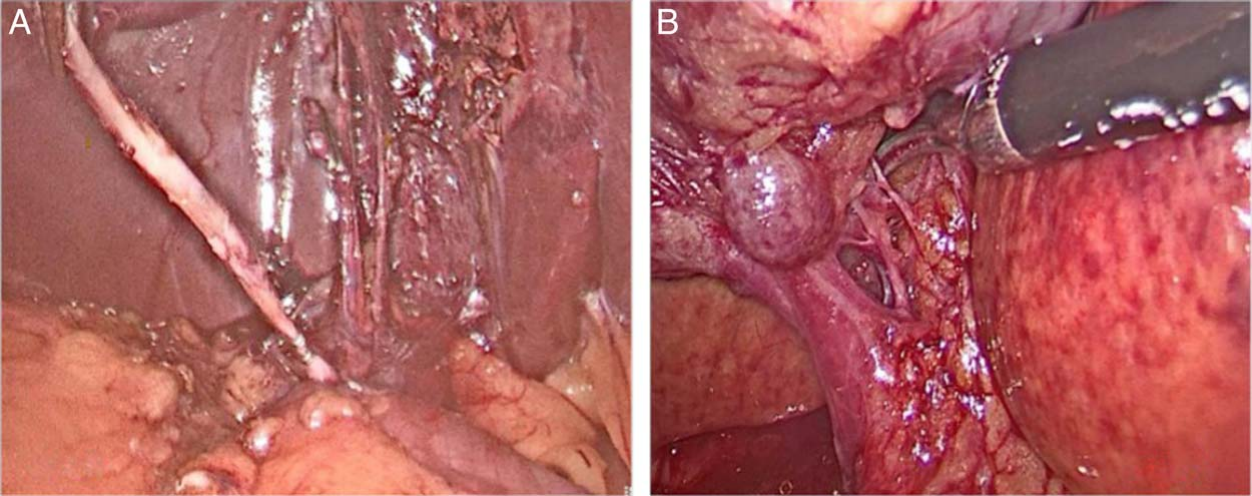
Intraoperative image of cystic duct showing long cystic duct (A) and short cystic duct (B) with multiple small cystic arteries.

**Figure 2:**
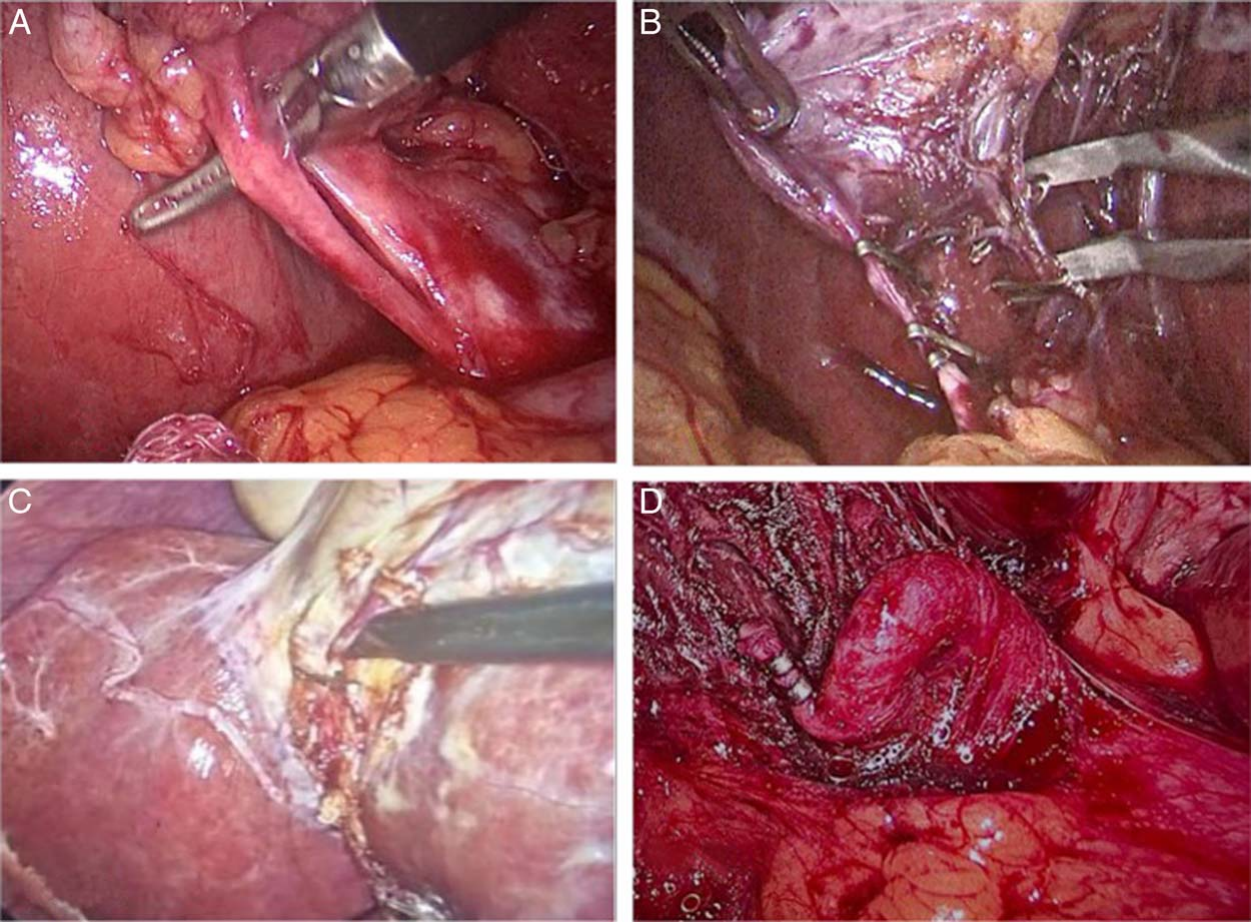
Intraoperative image of cystic artery showing anterior cystic artery (A), double cystic artery (B), artery originating from liver parenchyma (C) and caterpillar hump (D).

**Figure 3:**
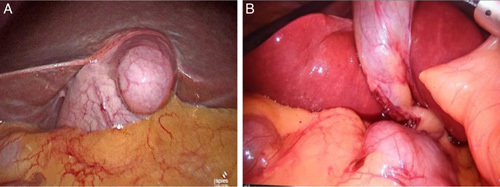
Intraoperative image of gall bladder showing diverticulum of the gall bladder (A) and left sided gall bladder (B).

The most common variations were found in the cystic artery (16.8%). CD anomalies were present in 11.4% of patients and gall bladder anomalies were the least common of all (5.4%). CD analysis revealed that 91.6% of patients had a normal length of the CD, 15 patients had a short CD (5%), and 10 patients had a long CD (3.4%) (Table 1). Abnormal insertion of the CD was found in 11 patients (3.7%). Two hundred and eighty five patients (95.6%) had an angular course of the CD, 10 patients (3.4%) had a parallel course and 3 patients (1%) had a spiral course. Long and short CD were the most frequently observed variations (Fig. 1). Cystic artery anomalies like anterior artery, multiple cystic artery, and caterpillar hump was observed in a few patients (Fig. 2). Cystic artery was found inside the hepatocystic triangle in 261 patients (87.6%). In 23 patients (7.7%) cystic artery was located outside the hepatocystic triangle, and 4 patients (1.3%) had a cystic artery in both in and out of the hepatocystic triangle. Two hundred and eighty two patients (94.6%) had a single cystic artery, 6 patients (2%) had a double cystic artery and in 10 cases the cystic artery was absent (3.4%). The cystic artery was present anterior to the CD in 32 patients (10.7%) and posteriorly in 256 patients (85.9%) (Table 2). In our study, the majority of patient had gall bladder at the normal site (97%), intrahepatic gall bladder was found in nine patients (3%). Rarer anomalies of the gall bladder include gall bladder diverticulum and left sided gall bladder (Fig. 3).

**Table 1:** Cystic duct and gall bladder anomalies.

Variables	Frequency	Percentage (%)
Site of gall bladder		
Normal	289	97
Intrahepatic	9	3
Number of gall bladder		
Single	298	100
Double	0	0
Other gall bladder anomalies^*^	7	2.3
Length of cystic duct		
Normal (2– 4 cm)	273	91.6
Short (<2 cm)	15	5.0
Long (>4 cm)	10	3.4
Number of cystic duct		
Single	295	99
Double	3	1
Insertion of duct		
Normal	287	96.3
Abnormal	11	3.7
Course of duct		
Angular	285	95.6
Parallel	10	3.4
Spiral	3	1

* Other Anomalies include two patients had Phrygian cap, three had diverticulum, one had septate gall bladder, one had left sided gall bladder.

**Table 2:** Anomalies of cystic artery.

Variables	Frequency	Percentage
Location of artery		
Inside hepatocystic triangle	261	87.6
Outside hepatocystic triangle	23	7.7
Both	4	1.3
Number of cystic artery		
Single	282	94.6
Double	6	2
Absent	10	3.4
Relation with cystic duct		
Anterior	32	10.7
Posterior	256	85.9
Others		
Caterpillar hump	3	1
Artery from parenchyma	2	0.7
Single large artery	2	0.7

### Intraoperative and postoperative complications

Out of 298 patients, intraoperative hemorrhage occurred in 20 patients (6.7%), intraoperative bile leak occurred in 28 patients (9.4%), and drain was inserted in 31 cases (10.4%). Three patients (1%) got converted from laparoscopic to open cholecystectomy. The proportion of patients with intraoperative hemorrhage was higher in anatomical variants as compared to normal patients (16.8% vs. 1.9%) (Table 3).

**Table 3:** Comparison of intraoperative and postoperative complications between patients with normal and abnormal anatomy.

Intraoperative and postoperative variables	Normal anatomy (*n*=203)	Anatomical Variants (*n*=95)	*P*
Intraoperative hemorrhage (*n*=20)	4	16	<0.001
Intraoperative bile leak (*n*=28)	13	15	0.01
Drain (*n*=31)	6	25	<0.001
Conversion to open (*n*=3)	0	3	0.03
VAS at 6 h	4.49±1.35	4.95±1.25	0.006
VAS at 24 h	2.6±1.32	3.09±1.14	0.002
VAS at 48 h	1.18±0.93	1.39±1.01	0.143
Length of stay	2.0 (1)	2.0 (1)	0.114
Port site infection	25	13	0.74

There was a statistically significant association between intraoperative hemorrhage and variations of the cystic artery, CD, and gall bladder, χ^2^(4) =49, *P*=<0.001. On subgroup analysis, the maximum association was between cystic artery anomalies and intraoperative hemorrhage with an adjusted residual value of 7.0. There was a strong association between the compared variables (Cramer’s V=0.409, φ =0.409, *P*=<0.001). The proportion of patients with intraoperative bile leak was higher in anatomical variants as compared to normal patients (15.7% vs. 6.4%). There was a statistically significant association between intraoperative bile leak and the presence of anatomical variations, χ^2^(1) =6.6, *P*=0.01.

On subgroup analysis, the maximum association was between gall bladder, CD anomalies, and intraoperative bile leak with adjusted residual value of 3.9 and 3.2, respectively. There was a strong association between the compared variables (Cramer’s V=0.314, φ =0.314, *P*=<0.001) (Table 4). There was a statistically significant association between conversion to open procedure and the presence of anatomical variations overall (*P*=0.03). Although on subgroup analysis, the difference was found to be statistically insignificant.

**Table 4:** Subgroup analysis of intraoperative and postoperative complications among gall bladder, cystic duct, and cystic artery anomalies.

Variables	Normal (*n*=203)	Cystic artery (*n*=46)	Cystic duct (*n*=32)	Gall bladder (*n*=12)	Mutiple (*n*=5)	*P*
Hemorrhage (*n*=20)	4	14	2	0	0	<0.001
Bile leak (*n*=28)	13	1	8	5	1	<0.001
Drain (*n*=31)	6	13	5	4	3	<0.001
Conversion to open (*n*=3)	0	2	1	0	0	0.04
VAS at 6 hours	4.4±1.3	5.0±1.2	4.9±1.2	4.5±1.2	5.8±1.3	0.02
VAS at 24 h	2.6±1.3	3.09±1.1	3.03±1.1	2.9±1.3	4.0±1.5	0.01
VAS at 48 h	1.18±0.9	1.4±0.9	1.5±1.08	0.67±0.8	1.7±0.9	0.1
Length of stay	1.8±0.7	2.02±0.88	2.0±0.9	2.0±0.8	1.6±0.5	0.5
Port site infection	25	6	5	1	1	0.9

We observed a statistically significant difference in the mean pain score of patients having normal anatomy (4.49±1.35) versus patients having anatomical variations (4.95±1.25), *P*=0.006 at 6 h and 24 h. However, the mean pain score at 48 h was not statistically significant. No significant difference was found between the length of hospital stay and port site infections.

## Discussion

Gall stones disease can occur at all ages, although it is most common in the 3rd and 4th decades of life. In our study of 298 patients, 50% of patients were in the 2nd and 3rd decade of life. Similar incidences were reported by Kumar *et al*.^10^, R Schmitz *et al*.^11^. Cholelithiasis is more common in the female sex, in our study, the majority of the patients were female (59.7%). Similar incidences are reported in other studies as well^9,12^. Earlier studies have found an increase rate of complications and conversion in patients with a higher BMI^13,14^. Few population-based studies have associated diabetes with an increased risk of gall stones and postoperative complications^15^. However, no significant difference was observed between the two groups in term of BMI and other comorbidities in our study (Table 5).

**Table 5:** Demographic and clinical profile of patients in both the groups.

Variables	Normal anatomy	Anatomical variations	*P*
Sex			
Female	124	54	0.487
Male	79	41	
Age			
<20 years	1	3	0.124^*^
20–50 years	135	66	
50 and above	67	26	
Socioeconomic status			
Upper	62	31	0.088
Middle	131	64	
Lower	10	0	
BMI			
Underweight	2	2	0.727^*^
Normal	71	35	
Overweight	52	27	
Obese	78	31	
Comorbidity			
DM	26	15	0.214
HTN	30	13	
Hypothyroid	8	9	
No comorbidity	139	58	

* Fisher’s exact test used.

In a previous study by Singh *et al*.^9^ on 800 patients, 650 cases had a normal single CD, which is consistent with the fact that CD variations are few when compared to vascular variations. A similar incidence was reported by shaw *et al*.^16^ Singh *et al*.^9^ reported a 2.6% incidence of the short CD. CD analysis in our study revealed, 15 patients had short CDs (5%), and 10 patients had long CDs (3.4%). These observed variations were less as compared to the reported incidence.

A long CD is advantageous to the surgeon because it allows for easy manipulation. A long, parallel, and spiral CD, on the other hand, could be dangerous because its end is unknown. Iatrogenic injury can result from mistaking the CD for the bile duct, such as inadvertent ligation or transection of the extrahepatic bile duct. An enlarged or long CD remnant may be associated with inflammatory changes and the formation of calculi, resulting in postcholecystectomy syndrome. A short CD poses difficulty while ligating the duct and at times extra traction on the gall bladder can cause the CD to come in line with the CBD, leading to incomplete or complete injury.

The path and pattern of entry of the CD vary greatly^17^, but it typically connects to the common hepatic duct below the confluence of the right and left hepatic duct on the lateral side in 58–75% of cases^18^. The remainder of it runs parallel to or around the hepatic duct before joining the bile duct. Talpur *et al*.^19^ found three common types of CD insertion: low CD insertion (9%), medial CD insertion (10–17%), and a parallel course of the CD with the common bile duct (1.25–5%). In our study, abnormal insertion of the CD was found in 11 patients (3.7%), the rest of the patients had normal insertion of the CD (96.3%). Two hundred and eighty five patients (95.6%) had an angular course of the CD, 10 patients (3.4%) had a parallel course and 3 patients (1%) had a spiral course.

Angular-type CD forms an acute angle with the common hepatic duct, parallel type runs parallel for varying distances before unifying. Spiral form, CD follows a spiral path before joining CHD on either the anterolateral or postero-lateral side. A parallel type of union can result in CD remnant syndrome. Indeed, parallel and spiral insertion of the CD poses significant difficulty in laparoscopic cholecystectomy.

Variations of the cystic artery are common. Singh *et al*.^9^ in their study found a high number of vascular anomalies (384 out of 740 patients). The most common anomaly of a cystic artery found in our study was the anterior cystic artery. The anterior cystic artery is always vulnerable to injury as it becomes the first structure to be encountered. The anterior cystic artery generally originates from the gastroduodenal artery and is low-lying. In conventional cholecystectomy, it generally lies inferior to the CD but in laparoscopic view, it becomes anterior to the CD. Caterpillar hump is another significant variation, because of the atypical course, the right hepatic artery comes into contact with the CD, resulting in the formation of a short cystic artery. As a result, the right hepatic artery may be misidentified as the cystic artery and accidentally ligated, resulting in torrential bleeding and, later, necrosis of the right lobe of the liver. Furthermore, because the cystic artery originating from the caterpillar hump is short, it is easily avulsed from the hepatic artery.

Gall bladder variations are uncommon and rarely encountered. In a study conducted by Dharmendra *et al*.^10^, found 6 out of 100 patients (6%) had gall bladder anomalies. Talpur *et al.* ^19^ in their study found six patients (2%) with gall bladder anomalies. Similarly, in our study, majority of patients had gall bladder at normal sites (97%).

Talpur *et al* ^19^ reported postoperative complications such as sepsis (7.33%), bleeding (3.67%), right shoulder pain (2.33%), and biliary leak (1.67%). In our study of 298 patients, intraoperative hemorrhage occurred in 20 patients (6.7%), intraoperative bile leak occurred in 28 patients (9.4%), drain was inserted in 31 cases (10.4%), 3 patients (1%) got converted from laparoscopic to open cholecystectomy. Subgroup analysis revealed a strong association between cystic artery anomalies and intraoperative hemorrhage. CD and gall bladder anomalies were associated with intraoperative bile leak.

### Limitations

This study has a few limitations. Observer bias cannot be completely ruled out as different surgeons have different perceptions of anatomical variations, although all the anatomical variations were recorded and reviewed by two different senior laparoscopic surgeons. Being an observational study comparison between normal individuals and patients with anatomical variations was limited, further trials are required for establishing a causal relationship. The use of intraoperative imaging in the form of an intraoperative cholangiogram or indocyanine green would have yielded better delineation of biliary anatomy, but this was not performed in this study.

## Conclusion

This study was conducted to estimate the prevalence of cystic artery, CD, and gall bladder anomalies in patients undergoing laparoscopic cholecystectomy. The study concluded with a significant prevalence of anatomical variations, with cystic artery anomalies being the commonest.

Patients with anatomical variations had significant intraoperative and postoperative complications compared to patients with normal anatomy. There was a strong association between intraoperative bleeding and cystic artery anomalies, and intraoperative bile leaks were associated with CD and gall bladder anomalies. As of now, preoperative imaging is not useful in detecting these variations, hence, we recommend more studies of this type with the incorporation of preoperative and intraoperative imaging modalities to better delineate these anatomical variations to prevent significant morbidity in the future.

## Ethical approval

This study is as per institutional ethics committee norm. The institute ethics committee approved this study, vide letter number AIIMS/Pat/IEC/2022/891.

## Consent

Written informed consent was obtained from patients for publication of this study and accompanying images. A copy of the written consent is available for review by the Editor-in-Chief of this journal on request.

## Sources of funding

None.

## Author contribution

A.K. and R.G.: conception or design of the work; R.G. and C.P.H.: data collection; R.G. and M.K.: data analysis and interpretation; A.K. and R.G.: drafting the article; A.K., M.K., and R.G.: critical revision of the article. All authors contributed in the final approval of the version to be published. The author(s) read and approved the final manuscript.

## Conflicts of interest disclosure

The authors declare that they have no financial conflict of interest with regard to the content of this study.

## Research registration unique identifying number (UIN)

1. Name of the registry: not applicable.
2. Unique Identifying number or registration ID: not applicable.
3. Hyperlink to your specific registration (must be publicly accessible and will be checked): not applicable.

## Guarantor

Dr Anil Kumar, Additional Professor (Gen Surgery), Head (Trauma Surgery & Critical Care)

All India Institute of Medical Sciences, Patna, India.

Type-5, Block-B, Flat No-104

AIIMS Residential Complex, Hydraulic Road, Khagaul 801105,

Patna, India. Tel: 91 9835699103.

E-mail: dranil4@gmail.com, dranilk@aiimspatna.org

## Provenance and peer review

Not commissioned, externally peer-reviewed.

## Data availability statement

The data that support the findings of this study are available from the corresponding author, Dr Anil Kumar, upon reasonable request.
